# Supplementary material for: Integrated Analysis of the Lung Microbiome and Metabolome Reveals Associations Between Amino Acid Metabolism and Pulmonary Fibrosis in a Bleomycin-Induced Mouse Model
Source: Int J Mol Sci. 2026 Jun 30;27(13):5895. doi: 10.3390/ijms27135895 (PMC13362081; doi:10.3390/ijms27135895)
Supplement: Supplementary file 1 [file ijms-27-05895-s001.zip › result/Result_list-Software_readme.pdf]

Result_list-Software_readme

1. **结果目录结构：**

|-- 1.MetQuant-QC【代谢物定性定量结果目录】

| |-- meta_intensity_{all}.{xls,xlsx} 【所有代谢物定性定量表】

| |-- Class_stat_{all}.xls 【代谢物的一级分类统计表】

| |-- Pie_Chart_{all}.{png,pdf} 【代谢物一级分类饼图】

| |-- sam_qc_infor_{all}.xls 【样本信息及总 PCA 图中样本编号信息】

| |-- Samples_QC_{all}-PCA[.3D].{png,pdf} 【QC 及所有代谢样本的总 PCA 图】

| |-- Samples_QC_pcaloading_{all}.{png,pdf} 【QC 及所有代谢样本总 PCA 图的载荷图】

| |-- Samples_{all}-PCA[.3D].{png,pdf} 【所有代谢样本的总 PCA 图】

| |-- Samples_pcaloading_{all}.{png,pdf} 【所有代谢样本总 PCA 图的载荷图】

| |-- **【TIC】**

| |-- {all} 【不同离子模式TIC图目录】

| | |-- *TIC.pdf 【QC 及样本的TIC 图】

| |-- **【Correlation】**

| |-- cor_pearson_{all}.{png,pdf,xls} 【QC 样本相关性分析结果】

|-- 2. MetAnnotation 【代谢物注释结果目录】

| -- **KEGG** 【基于 KEGG 数据库注释代谢物通路结果】

| |--meta_{all}_kegg_anno_category.xls 【KEGG 通路注释结果】

| |--meta_{all}.KEGG.Anno.{png,pdf} 【KEGG 注释结果统计图】

| |---meta_{all}.KEGG.anno.{xls,xlsx} 【代谢物 KEGG 注释结果列表】

| -- **HMDB** 【基于 HMDB 数据库注释代谢物分类结果】

| |--meta_{all}_hmdb_anno_category.xls 【HMDB 分类注释结果】

| |--meta_{all}.HMDB.Anno.{png,pdf} 【HMDB 分类注释结果统计图】

| |-- meta_{all}.HMDB.anno.{xls,xlsx} 【代谢物 HMDB 注释结果列表】

| -- **Lipidmaps** 【基于 Lipidmaps 数据库注释代谢物分类结果】

| |--meta_{all}_lipidmaps_anno_category.xls【Lipidmaps 分类注释结果】

| |--meta_{all}.Lipidmaps.Anno.{png,pdf}【Lipidmaps 分类注释结果统计图】

| |-- meta_{all}.Lipidmaps.anno.{xls,xlsx} 【代谢物 Lipidmaps 注释结果列表】

| -- **HMDB_KEGG_Lipidmaps** 【基于 KEGG、HMDB、Lipidmaps 数据库注释整合结果】

| -- meta_intensity _{all}_hmdb_kegg_lipidmaps.{xls,xlsx} 【总 HMDB、KEGG、Lipimaps 数据库注释整合结果】

|-- 3.MetDiffScreening 【差异代谢物筛选结果目录】

| -- *.vs.* 【样本比较对目录】

| |-- *.vs.*_{all}.{xls,xlsx} 【样本比较对定量分析结果】

| |-- *.vs.*_{all}_Diff_order.{xls,xlsx} 【样本比较对差异代谢物分析】

| |-- *.vs.*_{all}_diff.anno.{xls,xlsx} 【样本比较对差异代谢物注释结果】

| |-- *.vs.*_{all}-PCA[.3D].{png,pdf} 【样本比较对 PCA 分析】

| |-- *.vs.*_{all}-PCA-pcaloading.{png,pdf} 【样本比较对 PCA loading 分析】

| |-- *.vs.*_{all}-PLSDA-{score,valid}.{png,pdf}【比较对 PLSDA 分析】

| |-- *.vs.*_{all}-PLSDA-loading.{png,pdf}【比较对 PLSDA loading 分析】

| |-- *.vs.*_{all}_stem.{png,pdf}【比较对火柴杆图分析】

| |-- *.vs.*_{all}.xls.volcano.{png,pdf} 【样本比较对火山图分析】

|-- 4.MetDiffAnalysis 【差异代谢物分析结果目录】
| -- Box_diff【总差异代谢物箱线图目录】
| |-- {all}【不同离子模式箱线图目录】
| | |--*_{all}_Box_plot.{png,pdf} 【不同组间差异代谢物箱线图】
| -- Violin_diff【总差异代谢物小提琴图目录】
| |-- {all}【不同离子模式小提琴图目录】
| | |--*_{all}_Violin_plot.{png,pdf} 【不同组间差异代谢小提琴图】
| -- Heatmap_diff 【总差异代谢物热图目录。注：仅一个比较对无此目录和对应结果】
| |--Diff_Heatmap_{all}{_cluster,cluster_detail}.{png,pdf}【总差异代谢物聚类热图】
| |--Diff_Heatmap_{all}{,_detail}.{png,pdf}【总差异代谢物不聚类热图】
| |--Diff_Heatmap_Name_{all}.{xls,xlsx} 【总差异代谢物热图分析列表】
| |--Diff_Heatmap_{all}.heatmap_scale.{xls,xlsx} 【总差异代谢物热图 scale 转换后列表】
-- Kmeans_diff 【总差异代谢物 Kmeans 分析目录】
| |-- {all}【不同离子模式 Kmeans 分析目录】
| | |--Diff_kmeans_subcluster_{all}.{xls,xlsx}【总差异代谢物 Kmeans 分析列表】
| | |--Diff_kmeans_subcluster【总差异代谢物 Kmeans 分析目录】
| | | |--subcluster_*.{xls}【Kmeans 分析中每个 subcluster 分析表格】
| | | |--kmeans_subcluster_plots.{png,pdf}【Kmeans 分析结果图】
| -- Venn_diff【基于比较对的差异代谢物 venn 图目录】
| |--Diff_Venn_{all}.{png,svg} 【差异代谢物 venn 图】
| |--Diff_Venn_{Intersection, Union}_{all}_addinfo.{xls,xlsx} 【差异代谢物 venn 图列表】
| -- *.vs.* 【比较的样本对目录】
| |-- *.vs.*_{all}.corr.{xls,xlsx} 【差异代谢物相关性列表】
| |-- *.vs.*_{all}_Pvalue.{xls,xlsx} 【基于相关性分析的 p 值列表】
| |-- *.vs.*_{all}_zscore.{xls,xlsx} 【差异代谢物 zscore 分析列表】
| |-- *.vs.*_{all}_cluster_heatmap[,_detail].{png,pdf} 【差异代谢物聚类热图】
| |-- *.vs.*_{all}_heatmap[._detail].{png,pdf} 【差异代谢物不聚类热图】
| |-- *.vs.*_{all}_corr.{png,pdf} 【差异代谢物相关性分析图】
| |-- *.vs.*_{all}_zscore.{png,pdf} 【差异代谢物 z-score 图】
| |-- *.vs. *_{all}_chord. {png,pdf} 【差异代谢物和弦图】
| |-- ROC_{all} 【差异代谢物 ROC 曲线图目录】
| | |-- *_{all}_ROC.pdf 【差异代谢物 ROC 曲线图】
|-- 5.MetKEGGEnrichment 【差异代谢物 KEGG 富集分析结果目录】
| -- *.vs.* 【比较的样品对目录】
| |-- *.vs.*_{all}_KEGG_classification.{xls,xlsx}【差异代谢物 KEGG 分类分析列表】
| |-- *.vs.*_{all}_KEGG_classification.{png,pdf} 【差异代谢物 KEGG 分类图】
| |-- *.vs.*_{all}.kegg_enrichment.{xls,xlsx}【差异代谢物 KEGG 富集结果列表】
| |-- *.vs.*_{all}.KEGG_Enrich.scatterplot.{png,pdf}【KEGG 富集气泡图】
| |-- *.vs.*_{all}_KEGG_map 【KEGG 通路图】
| |-- *.vs. *_{all}.KEGG_net.{png,pdf}【差异代谢物 KEGG 调控网络图。注：如果信息搜集表没有填写“KEGG 物种缩写”，则无此结果】

| |-- *.vs. *_{all}.KEGG_net.xls 【差异代谢物 KEGG 调控网络结果列表。注：如果信息搜集表没有填写“KEGG 物种缩写”，则无此结果】

| |-- *.vs. *_ {all}.KEGG_net_id_all.xls 【差异代谢物 KEGG 调控网络互作关系列表。注：如果信息搜集表没有填写“KEGG 物种缩写”，则无此结果】

|-- 6. MetGSEA **【GSEA 富集分析】**

| -- *.vs.* 【比较的样品对目录】

| |-- {all} 【不同离子模式目录】

| | |-- *.vs.*_{all}_KEGG_gsea_result.{xls,xlsx} 【 GSEA 富集结果】

| | |--enplot_MAP*.png 【 GSEA 富集结果展示图】

| | |--MAP*_. {xls,xlsx} 【GSEA 富集条目详细结果（默认取 NSE 值最大 20 个和最小 20 个条目）】

2. 分析过程使用的软件版本汇总

分析	内容	软件	版本
**代谢物定性定量**	代谢物定性	Python	Python-3.5.0
	代谢物定量		
	代谢物分类	R	R-3.4.3
**数据质控**	QC 样本质控	R	R-3.4.3
	总样品 PCA 分析		
**代谢物注释**	KEGG\HMDB\LIPIDMAPS	python<br>R	Python-2.7.6<br>R-3.4.3
**差异分析**	主成分分析（PCA）	Python<br>R	Python-3.5.0<br>R-3.4.3
	偏最小二乘法判别分析（PLS-DA）		
	Venn 图分析		
	聚类分析		
	相关性分析		
	火山图分析		
	火柴杆图分析		
	小提琴图分析		
	K-Means 分析		
	箱线图分析		
	和弦图分析		
	Z-score 分析		

**KEGG 分析**	KEGG 分类	python<br>R	Python-3.5.0<br>R-4.0.3
	KEGG 富集		
	KEGG 调控网络图		
**GSEA 分析**	GSEA-KEGG 富集	GSEA	gsea-3.0
备注	所有分析内容， 数据处理主要用 Python，图表绘制用 R 语言		
提示	建议使用 Microsoft Office 软件打开表格，其他软件可能出现乱码。 由于 xls 格式文件对于某些特殊字符可能无法显示， 因此对于包含代谢物名称的 xls 格式文件提供对应的 xlsx 格式文件，两者内容没有区别，xls 格式文件更适合作图， xlsx 格式文件更适合阅读。		
